# Eicosanoids and Respiratory Viral Infection: Coordinators of Inflammation and Potential Therapeutic Targets

**DOI:** 10.1155/2012/236345

**Published:** 2012-05-15

**Authors:** Mary K. McCarthy, Jason B. Weinberg

**Affiliations:** ^1^Department of Microbiology and Immunology, University of Michigan, Ann Arbor, MI 48109, USA; ^2^Department of Pediatrics and Communicable Diseases, University of Michigan, Ann Arbor, MI 48109, USA

## Abstract

Viruses are frequent causes of respiratory infection, and viral respiratory infections are significant causes of hospitalization, morbidity, and sometimes mortality in a variety of patient populations. Lung inflammation induced by infection with common respiratory pathogens such as influenza and respiratory syncytial virus is accompanied by increased lung production of prostaglandins and leukotrienes, lipid mediators with a wide range of effects on host immune function. Deficiency or pharmacologic inhibition of prostaglandin and leukotriene production often results in a dampened inflammatory response to acute infection with a respiratory virus. These mediators may, therefore, serve as appealing therapeutic targets for disease caused by respiratory viral infection.

## 1. Respiratory Viruses

Viruses are the most frequent cause of respiratory infection in humans. It has been estimated that viruses cause up to 90% of lower respiratory infection (LRI) hospitalizations in children less than 5 years of age and up to 40% of hospitalizations in children age 5–18 years [1]. Among the most common causes of viral respiratory infection in children and adults are respiratory syncytial virus (RSV), influenza, rhinovirus (RV), adenovirus, parainfluenza virus (PIV), and human metapneumovirus (hMPV) [2]. Viral respiratory infection also causes substantial disease burden in the elderly and immunocompromised populations [3, 4].

The host immune system faces the task of effectively clearing a virus while limiting local tissue damage and inflammation. The immune response to viruses can be protective, aiding with clearance of virus from the lungs and resolution of disease caused by viral replication. Disease associated with respiratory viruses can also be caused by immune-mediated pathology. Virus-induced inflammation can be detrimental to the host, causing symptoms during acute infection and leading to damage that contributes to long-term residual lung disease. Eicosanoids are potent lipid mediators that play a role in many biological processes, including inflammation and immune function. Two classes of eicosanoids, the prostaglandins (PGs) and leukotrienes (LTs), have been increasingly studied in the context of respiratory viral infection. Because of these effects, eicosanoids are likely to make significant contributions to the pathogenesis of respiratory virus infection.

## 2. Eicosanoid Synthesis

### 2.1. Prostaglandins

**Figure 1:**
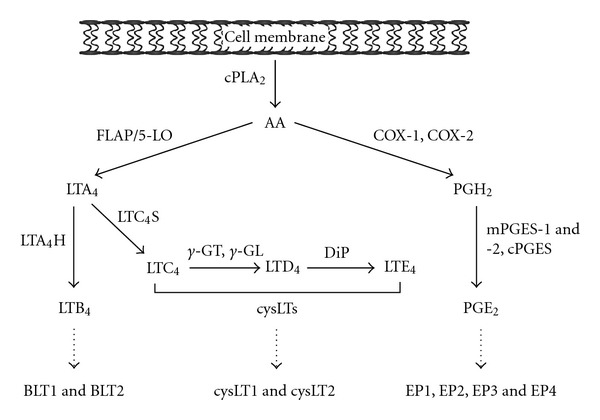
Synthesis of PGE_2_ and the leukotrienes. cPLA_2_—cytosolic phospholipase A_2_, AA—arachidonic acid, FLAP—5-lipoxygenase activating protein, 5-LO—5-lipoxygenase, LTA_4_—leukotriene A_4_, LTA_4_H—leukotriene A_4_ hydrolase, LTB_4_— leukotriene B_4_, BLT1 and BLT2—B leukotriene receptor 1 and 2, LTC_4_S—leukotriene C_4_ synthase, LTC_4_—leukotriene C_4_, *γ*-GT-*γ*-glutamyl transpeptidase, *γ*-GL-*γ*-glutamyl leukotrienease, LTD_4_—leukotriene D_4_, DiP—dipeptidase, LTE_4_—leukotriene E_4_, cysLTs—cysteinyl leukotrienes, cysLT1 and cysLT2—cysteinyl leukotriene receptor 1 and 2, COX-1 and COX-2—cyclooxygenase 1 and 2, PGH_2_—prostaglandin H_2_, mPGES-1 and -2—microsomal prostaglandin E synthase-1 and -2, PGE_2_—prostaglandin E_2_, EP1-4—E prostanoid receptors 1–4.

PGs are generated when phospholipase A_2_ (PLA_2_) releases arachidonic acid (AA) from membrane glycerophospholipids (Figure 1). Released AA is oxidized to the intermediate prostaglandin H_2_ (PGH_2_) by cyclooxygenase (COX). COX exists in three isoforms. COX-1 is generally constitutively expressed, while COX-2 expression is rapidly induced by growth factors and cytokines [5]. COX-3 is a recently discovered isoform whose biological role, if any, remains poorly understood [6, 7]. Once formed, PGH_2_ can be converted by specific synthases to thromboxane A_2_ (TXA_2_), PGD_2_, PGE_2_, PGF_2_, and PGI_2_. As described below, PGE_2_ has multiple effects on host immune function. PGE_2_ is transported from the cell by multidrug resistance protein (MRP) 4 and possibly by other unknown transporters [8]. The effects of PGE_2_ are mediated by its signaling through four distinct G protein-coupled E prostanoid (EP) receptors, EP1-4. The EP1 receptor is coupled to an unidentified G protein and mediates PGE_2_-induced increases in intracellular Ca^2+^ [9]. The EP2 and EP4 receptors mediate increases in cyclic AMP (cAMP) concentration by coupling to G*α*_*s*_. Four isoforms of the EP3 receptor are coupled to different G proteins, although the major EP3 receptor signaling pathway involves adenylate cyclase inhibition via G*α*_*i*_ coupling with subsequent decreases in intracellular cAMP [10]. The EP2 and EP4 receptors are expressed in almost all mouse tissues, while expression of EP1 is restricted to several organs, including the lung. EP2 expression is the least abundant of the EP receptors, however, several stimuli induce expression of EP2 [10].

### 2.2. Leukotrienes

LTs are also generated by liberation of AA from cell membranes (Figure 1). This is modified by a series of enzymes beginning with 5-lipoxygenase (5-LO), which acts in concert with 5-LO-activating protein (FLAP) to form leukotriene A_4_ (LTA_4_) [11]. LTA_4_ can then be metabolized by LTA_4_ hydrolase to form leukotriene B_4_ (LTB_4_). Alternatively, LTA_4_ can be conjugated to reduced glutathione by leukotriene C_4_ (LTC_4_) synthase to form LTC_4_. LTC_4_ is exported from the cell by specific transporters [12] and can be acted on by extracellular peptidases to form LTD_4_ or LTE_4_. Leukotrienes C_4_, D_4_, or E_4_ are collectively known as the cysteinyl leukotrienes (cysLTs).

Expression of 5-LO is tightly regulated and is primarily restricted to cells of the myeloid lineage, such as monocytes/macrophages, mast cells, eosinophils, and neutrophils. Although LT synthesis was once thought to be restricted to leukocytes, it has subsequently been shown that human bronchial epithelial cells and fibroblasts are capable of producing both cysLTs and LTB_4_ [13, 14]. In addition, the intermediate LTA_4_ can be transferred from an activated donor cell to a recipient cell. LTA_4_ can then be metabolized to either LTB_4_ or LTC_4_ by LTA_4_ hydrolase or LTC_4_ synthase, respectively, in a process termed “transcellular biosynthesis” [15]. These enzymes are expressed in most tissues. In this way, other cell types, such as epithelial cells, can become an important source of LTs during an inflammatory response.

Like PGE_2_, the effects of LTs are mediated by signaling through G protein-coupled receptors. Among the receptors for cysLTs, two have been thoroughly characterized. The cysLT1 receptor binds LTD_4_ with high affinity and binds LTC_4_ and LTE_4_ with lower affinities [16]. The cysLT2 receptor binds LTC_4_ and LTD_4_ with equal affinity. A number of studies have alluded to the existence of additional cysLTR subtypes, although these have yet to be characterized [17]. The chemoattractant and proinflammatory effects of LTB_4_ are mediated by the high-affinity B leukotriene receptor 1 (BLT1). A second receptor, B leukotriene receptor 2 (BLT2), binds LTB_4_ with lower affinity, but its biological function remains poorly understood [18]. Studies in transfected cell lines have shown that the four LT receptors can couple to both G*α*_i_ and G*α*_q_ proteins to decrease cAMP and increase intracellular Ca^2+^, respectively [19–23]. Studies in primary cells have yielded differing results and the specific signaling programs initiated by GPCRs remain to be dissected [24]. Within the human lung, cysLT1 mRNA is expressed in epithelial cells, bronchial smooth muscle cells, interstitial macrophages, and the nasal mucosa. CysLT2 is expressed by bronchial smooth muscle cells, interstitial macrophages, and nasal mucosa [17]. Human BLT1 is expressed primarily in leukocytes and its expression can be altered in response to various inflammatory stimuli [18, 25]. BLT2 is expressed more ubiquitously, with high mRNA expression detected in the spleen and low levels in most human tissues, including the lung [26].

## 3. Eicosanoids and Immune Function

### 3.1. Prostaglandin E_2_

PGE_2_ regulates immune function in a myriad of ways that are likely to affect viral pathogenesis (Table 1). Widespread expression of COX-2 has been demonstrated in airway epithelial and resident inflammatory cells in the absence of overt inflammation, suggesting a role for COX-2 in regulation of human airway homeostasis [27]. High concentrations of COX products are present in the epithelial lining fluid of human airways, potentially playing a role in inhibiting lymphocyte activity and fibroblast proliferation in the absence of inflammation [28]. Additionally, constitutive secretion of PGE_2_ by airway epithelial cells contributes to modulation of DCs under homeostatic conditions [29]. PGE_2_ can promote inflammation through vasodilatory mechanisms, yielding edema, warmth, erythema, and passive leukocyte recruitment. However, PGE_2_ is also capable of inhibiting neutrophil chemotaxis, phagocytosis, and bacterial killing [30, 31]. PGE_2_ suppresses phagocytosis by non-alveolar monocytes/macrophages [32–35], and PGE_2_ inhibits alveolar macrophage (AM) phagocytosis via a mechanism that involves EP2 activation and increases in cAMP [36]. Bacterial killing and reactive oxygen intermediate generation by AMs is also inhibited by PGE_2_ in an EP2/EP4- and cAMP-dependent manner [37].

The production of various pro-inflammatory cytokines and chemokines is inhibited in the presence of PGE_2_ [38, 39], while production of the anti-inflammatory cytokine interleukin (IL)-10 is enhanced [40]. PGE_2_ suppresses production of the Th1 cytokines interferon (IFN)-*γ* and IL-12, leading to a Th2-polarized environment [41, 42]. However, a number of studies have also reported PGE_2_-mediated enhancement of Th1 cytokine secretion and differentiation *in vivo *[43, 44]. The role of PGE_2_ is not strictly suppressive, as it has been shown to promote certain pathways in immune differentiation. For example, PGE_2_ can act on uncommitted B lymphocytes to promote isotype switching to IgE or IgG1 [45–47]. COX inhibitors inhibit antibody production in activated human B lymphocytes [48, 49]. PGE_2_ augments IL-17 production and Th17 differentiation by increasing IL-23 production in T cells and dendritic cells [44, 50–53], an activity that likely occurs via EP2- and EP4-mediated increases in cAMP [54, 55]. Additionally, PGE_2_ enhances the production of the proinflammatory cytokine IL-6 by leukocytes [56] and airway epithelial cells [57]. PGE_2_ potently inhibits the production of a number of antimicrobial peptides (AMPs) such as human *β*-defensin by epithelial cells [58]. This effect of PGE_2_ is likely to be relevant for viral pathogenesis, because AMPs can inhibit the replication of viruses [59, 60].

### 3.2. Leukotrienes

The diverse effects of LTs on innate immunity have been reviewed elsewhere [61] and are briefly summarized in Table 1. LTB_4_ promotes neutrophil migration and survival [62, 63] and enhances neutrophil granule enzyme secretion [64] and superoxide anion generation [65, 66]. T lymphocyte recruitment to sites of inflammation can be induced by LTB_4_ [67–70]. In addition to neutrophil and T cell trafficking, LTB_4_ can promote the migration of dendritic cells (DCs) *in vitro *[71] and to draining lymph nodes as mice deficient in BLT1/2 show reduced DC migration [72]. Both cysLTs and LTB_4_ can enhance Fc*γ* receptor-mediated phagocytosis by AMs, though by different mechanisms [24, 73, 74]. LTB_4_ induces antimicrobial peptide release from neutrophils *in vivo, *in some cases inhibiting viral replication [75–77]. Lung generation of the proinflammatory cytokine TNF-*α* is enhanced by LTB_4_ [78]. A number of studies have reported that LTB_4_ acts synergistically with IL-4 to induce activation, proliferation, and differentiation of human B lymphocytes [79–81], although a separate study reported that 5-LO inhibitors actually enhanced B lymphocyte proliferation [82].

CysLTs can promote microvascular leak [11], enhance leukocyte survival [83, 84], and induce nitric oxide (NO) generation in neutrophils [66, 85]. CysLTs induce DC chemotaxis to CCL19 and DC trafficking to lymph nodes is impaired in LTC_4_ transporter-deficient mice [12]. In addition, cysLTs have been suggested to play a role in allergen-induced DC migration from blood [86]. Addition of LTD_4_ to activated B lymphocytes leads to a modest upregulation of IgE and IgG production [87]. CysLTs also play a role in regulation of a pulmonary Th2 response as mice deficient in LTC_4_ synthase showed reduced Th2 cytokine mRNA expression and Ag-specific IgE and IgG1 in the lung [88]. CysLTs are recognized as important mediators in the pathogenesis of asthma by their ability to promote airway microvascular permeability, mucus secretion, and smooth muscle contraction [89–93].

The prostaglandins and leukotrienes modulate many host immune responses that are important contributors to viral pathogenesis, such as cytokine signaling, neutrophil and macrophage phagocytosis, trafficking and activation of DCs and T cells, and antibody production by B cells.

## 4. Eicosanoids and Respiratory Viruses

### 4.1. Influenza

Influenza infections account for over 200,000 hospitalizations annually in the USA [94]. In addition to hospitalizations, influenza is also associated with a substantial number of outpatient visits each year, causing considerable healthcare burden and economic costs. Influenza upregulates COX-2 expression both *in vitro *and *in vivo,* and it has been suggested that COX hyperinduction contributes to the exaggerated cytokine response observed in severe human H5N1 infections [95–97]. Alteration of the COX pathway has contrasting effects on inflammatory responses to influenza virus depending on the model of pharmacologic inhibition (COX-1- or COX-2-specific or dual inhibition) or of genetic deficiency. Treatment of influenza-infected mice with celecoxib, a selective COX-2 inhibitor, did not significantly affect viral titers or disease severity, although treatment did suppress production in the lung of the proinflammatory cytokines tumor necrosis factor- (TNF-) *α*, IL-6 and granulocyte-colony stimulating factor (G-CSF) [98]. In contrast, influenza infection of mice genetically deficient in COX-2 resulted in reduced mortality, inflammation, and cytokine responses compared to infection of wild-type control [99]. Peak lung viral titers were significantly elevated in COX-2^−/−^ mice but returned to levels seen in wild-type mice by day 6, suggesting a role for COX-2 in controlling early viral replication but not in virus clearance. Interestingly, levels of PGE_2_ in influenza-infected COX-2^−/−^ mice were equivalent to levels measured in infected wild-type mice. The lack of PGE_2_ deficiency in COX-2^−/−^ mice could be due to compensatory upregulation of COX-1 activity, as has been described before [100].

Mice infected with highly virulent H5N1 and treated with a combination of celecoxib, the neuraminidase inhibitor zanamivir, and mesalazine (an aminosalicylate drug that exhibits weak 5-LO and COX inhibition [101]) showed significantly improved survival even when treatment was delayed 48 hours [102]. The beneficial effect of celecoxib and mesalazine likely stemmed from their effects on immunopathology, as mice treated with triple therapy had similar viral loads as those treated with zanamivir alone. Triple therapy significantly reduced levels of the proinflammatory cytokines IL-6, TNF-*α*, and IFN-*γ*.

Another group treated influenza-infected mice with paracetamol (acetominophen), a selective inhibitor of COX-2 [103, 104]. Paracetamol-treated mice had improved lung function, and reduced immunopathology compared to control mice. A separate group of mice treated with celecoxib also showed improvements in cellular infiltrates, lung function and pathology. However, the degree of improvement was generally less than that seen in paracetamol-treated mice. In contrast to mice genetically deficient in COX-2 [99], paracetamol- and celecoxib-treated mice had viral loads equivalent to those in untreated control mice. Virus-specific CD4^+^ and CD8^+^ T cell numbers were not altered in treated mice, and treatment with paracetamol or celecoxib did not interfere with the establishment of protective immunity to a second infection with a different influenza subtype.

The significantly increased viral titers seen in COX-2^−/−^ mice but not observed in mice treated with COX-2 inhibitors could be due to a functional defect in innate immunity, as COX products are known to be involved in modulating the innate immune response [105]. In addition, COX-2^−/−^ mice have a complete loss of COX-2 activity, whereas mice treated with inhibitors still retain some COX-2 activity due to insufficient inhibition by the drug. COX-2^−/−^ mice had levels of PGE_2_ in bronchoalveolar lavage (BAL) fluid similar to wild-type mice, suggesting that the effects of COX-2 deficiency in this model may not be due to lack of PGE_2_. As COX-2^−/−^ deficiency is likely to affect the production of other prostaglandins (such as TXA_2_, PGD_2_, PGF_2_, and PGI_2_), it is possible that decreased levels of one of the other COX products are responsible for increased survival.

Influenza infection upregulates 5-LO expression and/or levels of LTs in cell lines as well as in lungs of infected mice and humans [106–108]. However, few studies have examined influenza infection in the context of altered 5-LO production (either due to genetic deficiency or pharmacologic inhibition). One study has reported a beneficial effect of exogenous LTB_4_ administration during influenza infection of mice [75]. Mice treated daily with LTB_4_ had significantly reduced lung viral loads. The lungs of LTB_4_-treated mice showed increased levels of multiple antimicrobial peptides, decreased inflammatory cell infiltration, and partially restored lung architecture. The antiviral effect of LTB_4_ was mediated by neutrophils and the high-affinity BLT1 receptor, as viral loads were unaffected in neutrophil-depleted or BLT1-deficient mice. LTB_4_ treatment of primary human neutrophils in this study induced antimicrobial peptide release and decreased influenza titers, demonstrating that the effects of LTB_4_ on neutrophils are similar in both mice and humans. This is in agreement with another study, in which human neutrophils treated with LTB_4_ showed significantly more myeloperoxidase (MPO) activity and *α*-defensin production than untreated cells, and LTB_4_-treated neutrophils had enhanced virucidal activity against influenza virus, human coronavirus, and RSV [109]. The role of cysLTs during influenza infection has yet to be defined in detail. Enhanced levels of cysLTs seen in COX-2^−/−^ mice infected with influenza are associated with increased survival [99], but whether the decreased mortality in COX-2-deficient mice is directly due to cysLTs in this model is unknown.

The beneficial effects of COX-2 deficiency may also be due to shunting of released AA to the 5-LO pathway. A number of reports suggest that COX inhibitors enhance production of LTs [110, 111]. Indeed, COX-2^−/−^ mice showed higher BAL fluid levels of cysLTs than wild-type mice following infection with influenza. However, in mice treated with a combination of zanamivir, celecoxib, and mesalazine, increased survival was associated with lower LT levels and higher PGE_2_ levels in the treated mice compared to wild type. The discrepancies in COX and 5-LO products in these models may reflect the different pathophysiology of the influenza strains used. Perhaps increased LT production during severe H5N1 infection promotes inflammation and local tissue damage, while PGE_2_ provides a balancing protective influence. In contrast, during infection with the less virulent H3N2 virus, enhanced LT production may contribute to virus clearance without a detrimental effect on host inflammation. However, in the case of either virus lower levels of the proinflammatory cytokines IL-6, TNF-*α*, and IFN-*γ* were correlated with decreased morbidity and increased survival. Other differences in the studies could be accounted for by differences in virus subtype, virus inoculum, mouse strain, or drug dose and delivery method. However, partial COX inhibition by pharmacologic intervention appears to be beneficial in reducing immunopathology while still controlling viral replication during influenza infection in mice.

### 4.2. Respiratory Syncytial Virus

Respiratory syncytial virus (RSV) is the leading cause of bronchiolitis and pneumonia in infants [112, 113]. RSV is also a significant pathogen in the elderly population, particularly those living in long-term care facilities or with underlying cardiopulmonary disease [114]. The immunocompromised are at risk for severe RSV infection, with mortality rates of up to 80% reported for RSV pneumonia [115]. RSV induces PGE_2_ release *in vitro, *in animal models, and in lungs of infants with RSV bronchiolitis [116–119]. Treatment with COX inhibitors reduces RSV replication *in vitro* and diminishes immunopathology *in vivo. *Blocking PG production with NS-398, celecoxib, or the cPLA_2_ inhibitor pyrrophenone reduced virus particle production in the A549 airway epithelial cell line [116]. COX inhibition also reduced transcription and production of the proinflammatory cytokines IL-8 and RANTES (CCL5). RSV-induced activation of interferon regulatory factor (IRF) and NF-*κ*B activation were suppressed by a high concentration of celecoxib. Another study demonstrated that the nonselective COX inhibitor indomethacin decreased lung histopathology in RSV-infected cotton rats, but COX inhibition did not significantly affect viral replication [117].

RSV also induces production of LTB_4_ and cysLTs in both animal models and infants afflicted with RSV bronchiolitis [119–125]. LT concentrations during RSV infection have been correlated with development of symptoms and in some reports are associated with disease severity [107, 122, 126, 127]. Treatment of RSV-infected mice with the 5-LO inhibitor zileuton reduced inflammatory cell numbers in the lung, prevented RSV-induced weight loss, and decreased RSV-induced airway constriction [122]. Viral titers were somewhat lower in the lungs of zileuton-treated mice, although the difference was not statistically significant. Even when administered after the emergence of respiratory symptoms, zileuton reduced airway resistance and weight loss compared to untreated mice. Treatment with the cysLTR1 antagonist MK-571 decreased RSV-induced airway hyperreactivity (AHR) [121]. In contrast to treatment with zileuton, MK-571 did not affect inflammatory cell recruitment or production of IL-4 and IFN-*γ* in RSV-infected mice. A possible effect of MK-571 on viral titers was not examined in this study.

Similar to highly virulent influenza H5N1, successful treatment of RSV infection may require the use of an antiviral agent in combination with an anti-inflammatory agent that limits immunopathology. In support of this, treatment of RSV-infected cotton rats with the RSV-specific humanized monoclonal antibody palivizumab and a glucocorticoid resulted in enhanced clearance of RSV and limited lung histopathology compared to controls [128]. Further support comes from a model of pneumonia virus of mice (PVM), a paramyxovirus that is a close phylogenetic relative of RSV. PVM infection increased levels of cysLTs in the lung [129]. In this model, administration of either the cysLT1 antagonist montelukast or the nucleoside analog ribavirin did not affect disease severity. However, combined therapy of montelukast with ribavirin substantially decreased morbidity and mortality of PVM-infected mice.

Administration of montelukast during primary RSV infection prevented enhanced AHR, airway eosinophil recruitment, and mucus overproduction upon reinfection [120]. Montelukast administered only during secondary infection did not affect this enhanced response. Previous studies have shown that LTs are only transiently elevated during the acute phase of infection and that levels drop to baseline shortly after [130]. This suggests that LT inhibitors may have a beneficial effect during the early phase of infection but may no longer be useful as treatment for the long-term airway dysfunction observed after RSV infection when LT levels are no longer elevated.

The above reports demonstrate a beneficial effect of 5-LO product inhibitors or cysLT1 receptor antagonists during primary infection with RSV. However, the studies in animal models used pharmacologic agents given to mice starting on the day before infection, whereas treatment in humans is typically initiated later during the course of infection after the emergence of symptoms. Delaying zileuton treatment until 3 days post infection, after respiratory symptoms emerged, still reduced clinical signs during primary RSV infection in mice. However, there have been conflicting results when 5-LO inhibitors and cysLT antagonists were used as treatment in children with RSV bronchiolitis. One study suggested a beneficial effect of the cysLTR1 antagonist montelukast on lung symptoms after RSV bronchiolitis [131], but further studies have failed to corroborate these findings [132–134]. To our knowledge, there are no human studies that examine prophylactic administration of 5-LO pathway inhibitors or receptor antagonists to high-risk children. Further studies are needed to define the role of LT inhibitors in patients with primary RSV infection and in those experiencing persistent airway dysfunction after RSV.

While many viruses are capable of causing respiratory infections, relatively little is known about the contributions made by eicosanoids to the pathogenesis of respiratory viruses other than influenza and RSV. Rhinovirus (RV) infection increases expression of 5-LO, FLAP, and COX-2 in human bronchial cells [135]. In addition, cysLT levels in BAL fluid are increased upon rhinovirus infection in humans and correlate with emergence of upper respiratory symptoms [107, 135]. Adenovirus induces COX-2 expression and PGE_2_ release in murine fibroblasts [136] and in human primary synovial fibroblasts [137]. Additional studies are necessary to examine adenovirus-induced PG production in lung-relevant cell types, but *in vivo* studies of human adenovirus pathogenesis are limited by the strict species specificity of adenoviruses. Using mouse adenovirus type 1 to study the pathogenesis of adenovirus respiratory infection [138] will provide a useful tool to define the roles of eicosanoids to adenovirus respiratory infection.

Human cytomegalovirus (HCMV) can also cause respiratory infections, although symptomatic disease is uncommon in immunocompetent individuals [139]. HCMV induces 5-LO expression and LTB_4_ production [140] in vascular smooth muscle cells as well as COX-2 expression and PGE_2_ production in fibroblasts [141]. COX-2 inhibition reduces levels of the immediate-early 2 mRNA and protein in addition to viral DNA replication and transcription of some early and late mRNAs. Treatment of HCMV-infected fibroblasts with COX inhibitors inhibits cell-to-cell spread of virus [142]. Of note, while many reports with other viruses have shown inhibition of viral replication or gene transcription by COX inhibitors at non-physiologic concentrations, these results with HCMV were obtained with concentrations of COX inhibitors that are achievable in human plasma. Although few studies have examined the effect of 5-LO products on HCMV pathogenesis, one study reported that exogenous LTB_4_ inhibited reactivation of CMV following allogeneic bone marrow transplantation (BMT) in mice, demonstrating a beneficial effect for this LT [143].

## 5. Common Themes

From the data summarized above (see also Table 2), it is clear that the effect of COX or 5-LO inhibition or antagonism of cysLT receptors on host responses to respiratory viral infection is variable and in some cases may be pathogen- and/or model-specific. In general, COX inhibition or deficiency is associated with less exuberant inflammation and in some cases improved survival. COX products may play a role in controlling early viral replication, although this possible role is only evident for influenza infection in mice completely lacking COX-2 activity and not in mice treated with a COX-2 inhibitor. These data are consistent with the role of PGE_2_ as an immunomodulatory mediator, balancing pro-inflammatory actions with suppressive effects on innate and adaptive immune function. Inhibition of LT production or signaling during respiratory viral infection is associated with less inflammation accompanied by variable (but generally beneficial) effects on lung physiology. However, administration of exogenous LTB_4_ also blunted inflammatory responses to influenza virus in one study [75], suggesting that various 5-LO products may be differentially involved in promoting inflammation and affecting host immune responses to viral infection.

## 6. Therapeutic Implications

Respiratory viral infections cause substantial disease and are associated with significant morbidity, mortality, and healthcare utilization. Many antiviral drugs are available to treat infection with human immunodeficiency virus, and a smaller number of drugs such as acyclovir and ganciclovir are available to treat infections with herpesviruses such as herpes simplex virus, varicella zoster virus, and HCMV. In contrast, far fewer drugs are available to treat viruses that most frequently cause respiratory infections. Neuraminidase inhibitors such as oseltamivir and zanamavir can be used as prophylaxis to prevent infection by influenza virus or used to treat infection. Older drugs such as amantadine and rimantadine can also be used to prevent or treat influenza. However, the emergence of drug-resistant influenza strains has the potential to increasingly limit the utility of these drugs. The nucleoside analog cidofovir has been used to treat adenovirus infections, although it has substantial toxicities and no randomized clinical trials have been performed to show clinical benefit. Currently, there are no drugs that have consistently been shown to be safe and effective for the treatment of disease caused by infection with RSV, rhinovirus, human metapneumovirus, or other viruses that commonly cause respiratory infections.

Preventing virus-induced inflammation may serve as an important adjunct to any antiviral therapy. When antiviral drugs are not available, modulation of virus-induced inflammation by itself may serve as an effective strategy to treat disease caused by viruses. Drugs with the ability to modulate eicosanoid production, such as ibuprofen and acetaminophen, are already frequently used in patients with respiratory infections to alleviate fevers, myalgias, and nonspecific symptoms. Studies described above that show decreased virus-induced inflammation and increased survival in animals treated with an inhibitor of PG or LT synthesis or in PG- or LT-deficient animals support the potential benefit of this approach. Drugs that modulate eicosanoid production may be particularly useful to prevent or treat infections in patients with exaggerated eicosanoid production at baseline. For instance, exaggerated PGE_2_ production in the setting of bone marrow transplantation has been associated with increased susceptibility to bacterial infection that is linked to impaired neutrophil and macrophage phagocytosis and killing [144, 145]. Increased PGE_2_ production has been reported in humans with a variety of disease states including cancer [146], aging [147], HIV infection [148], malnutrition [149, 150], and stem cell and solid organ transplant recipients [151, 152], making the potential benefits of this approach more widespread.

Any therapy that involves modulation of eicosanoid production must consider the potential for deleterious effects on the development of adaptive immunity and subsequent protection from secondary infection. PGE_2_ plays an important role in optimal antibody synthesis, as COX inhibitors reduce antibody production in activated human B lymphocytes [48, 49]. In addition, mice genetically deficient in COX-2 produce significantly less IgM and IgG than wild-type mice [48]. There is evidence that COX-2 plays a role in potentiating antibody production in humans as well. Human volunteers challenged with RV showed increased nasal symptoms and a suppressed serum neutralizing antibody response when treated with aspirin or acetaminophen, suggesting a protective role for COX products in reducing symptoms and promoting an antibody response [153]. One large-scale study has been performed in which children were administered prophylactic paracetamol when receiving routine childhood vaccinations [154]. Antibody responses to several of the vaccine antigens were less robust in patients receiving prophylactic paracetamol. Evidence also exists that LTs, like PGE_2_, promote appropriate antibody responses [79–81, 87], but the effect of 5-LO inhibitors and receptor antagonists on antibody production has not yet been described.

## 7. Conclusions

Eicosanoids modulate many host immune responses that are important contributors to viral pathogenesis. It will be essential to better define mechanisms underlying the effects of eicosanoids on both innate and adaptive immune responses to respiratory viral infection in order to develop therapies with maximal anti-inflammatory benefit and minimal impact on protective immune responses. For instance, the use of specific receptor agonists or antagonists may eventually provide a better-tailored approach than inhibitors of PG or LT synthesis to treat patients with respiratory viral infections. In general terms, however, alteration of eicosanoid production or antagonism of eicosanoid receptors has the potential to serve as a useful treatment strategy for respiratory viral infections.

## Figures and Tables

**Table 1:** Effects of PGE_2_ and leukotrienes on immune function.

	PGE_2_	LTB_4_	cysLTs
Neutrophils	Inhibits neutrophil chemotaxis, phagocytosis, and bacterial killing	Promotes neutrophil chemotaxis, ROS generation, and survival	Induces NO generation in neutrophils

Macrophages	Inhibits AM phagocytosis, ROS generation, and bacterial killing	Enhances AM phagocytosis	Enhance AM phagocytosis

T cells	Promotes Th17 differentiation	Induces T cell recruitment	Enhances Th2 response

B cells/Antibody production	Promotes isotype switching to IgE and IgG1	Induces activation, differentiation, and proliferation of B cells	Upregulate IgE and IgG1 production by B cells

Dendritic Cells	Varies	Promotes DC migration	Promotes DC migration

Cytokines	Suppresses IFN-*γ* and IL-12 production	Enhances TNF-*α* production	Enhances IL-5, IL-13, and eotaxin expression
	Enhances IL-10 and IL-6 production		

Antimicrobial Peptides	Inhibits AMP production by epithelial cells	Induces AMP production by neutrophils	Unknown

**Table 2:** Effects of PGE_2_ and Leukotrienes on respiratory syncytial virus and influenza infection.

	PGE_2_		Leukotrienes
	COX Inhibition	COX-2 Deficiency	
	Reduction in viral replication *in vitro *		Reduction in pulmonary inflammatory, weight loss, and RSV-induced airway constriction in mice treated with 5-LO inhibitor
RSV	Suppression of virus-induced cytokine production in vitro		
	No effect on viral replication in the lungs *in vivo *		CysLTR1 antagonism during primary infection prevents enhanced AHR upon reinfection
	Decreased lung pathology *in vivo *		Decreased RSV-induced AHR but no effect on cytokine production in mice treated with cysLTR1 antagonist

	No effect on viral replication or disease severity in micetreated with celecoxib	Decreased mortality, pulmonary inflammation and cytokine responses in COX 2^–/–^ mice	Reduced lung viral loads and decreased pulmonary inflammatory in mice treated with exogenous LTB_4_
Influenza	Suppression of virus-induced cytokine production in mice treated with celecoxib	Increased viral titers in lungs of COX-2^−/−^ mice compared to controls
	Improved survival and reduced proinflammatory cytokine levels in mice treated with zanamivir, celecoxib, and mesalazine	
	Improved lung function and reduced immunopathology in mice treated with paracetamol	
